# Incident dementia in ischaemic stroke patients with early cardiac complications: A propensity-score matched cohort study

**DOI:** 10.1177/23969873241293573

**Published:** 2024-11-02

**Authors:** Tommaso Bucci, Sylvia E Choi, Christopher TW Tsang, Kai-Hang Yiu, Benjamin JR Buckley, Pasquale Pignatelli, Jan F Scheitz, Gregory YH Lip, Azmil H Abdul-Rahim

**Affiliations:** 1 Liverpool Centre for Cardiovascular Science at University of Liverpool, Liverpool John Moores University and Liverpool Heart & Chest Hospital, Liverpool, UK; 2 Department of Cardiovascular and Metabolic Medicine, Institute of Life Course and Medical Sciences, University of Liverpool, Liverpool, UK; 3 Department of Clinical Internal, Anesthesiologic and Cardiovascular Sciences, Sapienza University of Rome, Rome, Italy; 4 Cardiology Division, Department of Medicine, The University of Hong Kong, Hong Kong, China; 5 Cardiovascular Health Sciences, Research Institute for Sport and Exercise Sciences, Liverpool John Moores University, Liverpool, UK; 6 Department of Neurology and Center for Stroke Research Berlin (CSB), Charité-Universitätsmedizin Berlin, Berlin, Germany; 7 Danish Center for Health Services Research, Department of Clinical Medicine, Aalborg University, Aalborg, Denmark; 8 Stroke Division, Department Medicine for Older People, Mersey and West Lancashire Teaching Hospitals NHS Trust, Prescot, UK

**Keywords:** Stroke, cardiovascular events, dementia

## Abstract

**Introduction:**

The risk of dementia in patients with stroke-heart syndrome (SHS) remains unexplored.

**Patients and methods:**

Retrospective analysis using the TriNetX network, including patients with ischaemic stroke from 2010 to 2020. These patients were categorised into two groups: those with SHS (heart failure, myocardial infarction, ventricular fibrillation, or Takotsubo cardiomyopathy within 30 days post-stroke) and those without SHS. The primary outcome was the 1-year risk of dementia (vascular dementia, dementia in other disease, unspecified dementia, or Alzheimer’s disease). The secondary outcome was the 1-year risk of all-cause death. Cox regression analysis after 1:1 propensity score matching (PSM) was performed to calculate the hazard ratios (HRs) and 95% confidence intervals (CIs) for the outcomes.

**Results:**

We included 52,971 patients with SHS (66.6 ± 14.6 years, 42.2% females) and 854,232 patients without SHS (64.7 ± 15.4 years, 48.2% females). Following PSM, 52,970 well-balanced patients were considered in each group. Patients with SHS had a higher risk of incident dementia compared to those without SHS (HR 1.28, 95%CI 1.20–1.36). The risk was the highest during the first 31 days of follow-up (HR 1.51, 95%CI 1.31–1.74) and was mainly driven by vascular and mixed forms. The increased risk of dementia in patients with SHS, was independent of oral anticoagulant use, sex and age but it was the highest in those aged <75 years compared to ⩾75 years.

**Discussion and conclusion:**

SHS is associated with increased risk of dementia. Future studies are needed to develop innovative strategies for preventing complications associated with stroke-heart syndrome and improving the long-term prognosis of these patients.

## Graphical abstract

**Graphical abstract:**
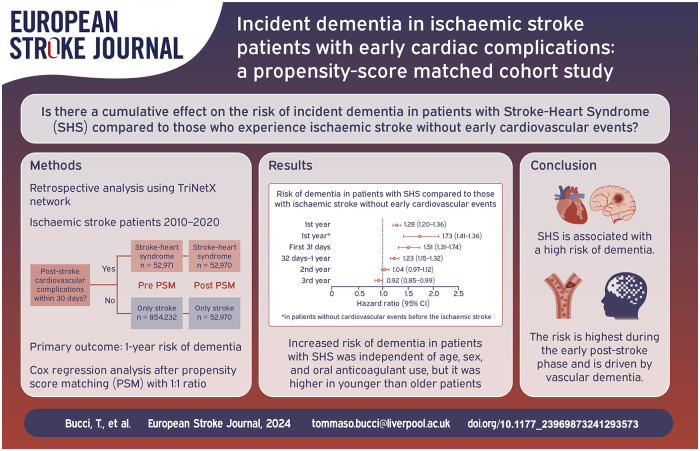

## Introduction

Patients with ischaemic stroke are at high risk for early cardiovascular complications, which are significantly associated with worsening morbidity and mortality.^1–3^ Neuronal injury post-stroke triggers the release of substantial amounts of catecholamines and cytokines, leading to a systemic inflammatory response coupled with impaired antioxidant systems that can result in a broad spectrum of cardiac complications.^2–5^

Stroke-heart syndrome (SHS) encapsulates the early cardiovascular complications following acute ischaemic stroke, characterised by the emergence of new cardiac conditions or the exacerbation of pre-existing cardiac diseases within 30 days of the stroke onset.^2,6^ It has been reported that approximately 25% of patients with ischaemic stroke develop early cardiovascular complications, with the highest incidence occurring within the first 3 days post-stroke.^2^ Recognised risk factors for SHS include advanced age, pre-existing cardiovascular conditions and specific stroke characteristics, such as stroke severity, infarct size and lesion location in the insular cortex.^7^ SHS can present with a broad spectrum of cardiovascular complications, ranging from subclinical manifestations like reduced heart rate variability or impaired baroreceptor reflex sensitivity to potentially life-threatening conditions such as new-onset acute myocardial infarction (AMI), heart failure (HF), atrial fibrillation, ventricular fibrillation or flutter (VFF) and Takotsubo cardiomyopathy (TTS).^6^ Previous studies have shown that the onset of SHS is associated with 2–3 times the risk of short-term mortality or poor functional outcomes and 1.5–2 times the risk of mortality and major adverse cardiovascular events within 5 years post-stroke, compared to patients without SHS.^8^

Recent evidence shows that both ischaemic stroke and cardiovascular events increase the risk of dementia.^9–12^ However, the potential cumulative effect of ischaemic stroke combined with early cardiovascular complications (SHS), on dementia risks remains unexplored. We hypothesised that ischaemic stroke patients with early cardiovascular complications as part of the SHS are at increased risk of incident dementia. To address this, we assessed the risk of incident dementia in patients with SHS compared to those without SHS in a global federated research database.

## Methods

### Study design

This study was a retrospective observational analysis carried out using TriNetX, a worldwide federated health research network with access to electronic medical records (EMRs) from various participating healthcare centres. These encompass academic medical centres, specialty physician practices and community hospitals, collectively covering an estimated 300 million individuals worldwide. Within this expansive network, accessible data encompass demographic details, diagnoses recorded using International Classification of Diseases, Ninth and Tenth Revisions, Clinical Modification (ICD-10-CM) codes, as well as medication information coded using Veteran Affairs (VA) Codes. Further details are available online at https://trinetx.com/.

TriNetX is a health research network compliant with the Health Insurance Portability and Accountability Act and the United States (US) federal law that safeguards the privacy and security of healthcare data, including de-identified data as per the de-identification standard of the HIPAA Privacy Rule. To gain access to the data in the TriNetX research network, requests are directed to TriNetX and a data sharing agreement is required. As a federated research network, studies using the TriNetX health research network do not need ethical approval as no patient identifiable information is received. Further information about the data extraction from TriNetX is reported in the Supplemental material.

### Cohort

The searches on the TriNetX online research platform were performed on the 14th of September 2024 for individuals aged ⩾18 years who experienced an ischaemic stroke between 1st January 2010 and 31st of December 2020. Based on the development of early cardiovascular complication (AMI, acute HF, VFF or TTS) within 30 days from the stroke, patients were categorised into two groups: patients with SHS and those without SHS (i.e. patients who experienced stroke only) (Supplemental Figure 1). More information about the ICD-10-CM codes utilised for the inclusion and exclusion criteria can be found in Supplemental Table 1.

At the time of the search, 93 participating healthcare organisations, primarily located in the US, had data available for patients who met the study’s inclusion criteria. Any other diagnoses or treatment reported prior to stroke onset were considered the individual’s baseline characteristics. Patients with a prior diagnosis of Alzheimer’s disease, vascular dementia, unspecified dementia, or dementia in other diseases classified elsewhere, as well as those who died within the first 30 days post-ischaemic stroke were excluded.

### Outcomes

The primary outcome was the 1-year risk of a composite of Alzheimer’s disease, vascular dementia, unspecified dementia and dementia in other diseases classified elsewhere. The secondary outcome was the 1-year risk of all-cause death. The adverse events of interest were identified via ICD-10-CM codes (Supplemental Table 2).

### Statistical analysis

Baseline characteristics of patients with SHS and those without SHS were balanced using logistic regression and propensity score matching (PSM) with a 1:1 ratio. The greedy nearest neighbour method with a caliper of 0.1 pooled standard deviations without replacement was applied. The balance of demographic and clinical variables between groups was evaluated using Absolute Standardised mean Differences (ASD), whit an ASD < 0.1 indicating well matched characteristics. The variables included in the PSM were age, sex, ethnicity, hypertension, diabetes, dyslipidaemia, obesity, chronic kidney disease, sleep apnoea, chronic ischaemic heart diseases, previous ischaemic or haemorragic stroke, chronic heart failure, atrial fibrillation, pulmonary embolism, peripheral artery disease and cardiovascular medications (such as β-blockers, antiarrhythmics, diuretics, lipid lowering agents, antianginals, calcium channel blockers, angiotensin-converting enzyme inhibitors, angiotensin II receptor blockers, oral anticoagulant (OAC) and antiplatelets). These variables were selected based on their potential association with the cardiovascular risk, supporting our hypothesis that early cardiovascular events in stroke patients may contribute additively to dementia risk. Subsequently, Cox proportional hazard models were used post-PSM to calculate hazard ratios (HRs) and 95% confidence intervals (95%CI) for the risk of defined outcomes in patients with SHS compared to those without SHS. Kaplan-Meier survival curves were constructed for the primary and secondary outcomes to illustrate differences in survival rates among groups. The Log-rank test tests for between-group differences in the probability of developing the outcome of interest at any time point within the study. The index event, marking the start of the observation period, was the 31st day after the ischaemic stroke. Follow-up time was calculated for each patient meeting the index criteria, representing the number of days between the index event and either the end of the analysis window or the patient’s last known data point. Follow-up time was reported as the median, with the interquartile range (IQR) calculated as the difference between the 75th and 25th percentiles of follow-up duration. Patients were censored when they no longer provided data for analysis.

To assess whether the proportional hazards assumption held in the Cox regression models, we applied a Chi-square (χ²) test based on Schoenfeld residuals. More information regarding the performance and interpretation of these test are provided in the Supplemental material. In cases where the proportional hazards assumption in the primary analysis was not met, we divided the 1-year follow-up period into two phases: an early phase (the first 31 days of follow-up) and a late phase (from day 32 day to the end of the first year). We then re-evaluated the risk using Cox regression and retested the proportional hazards assumption for each phase.

The competitive risk analyses were performed utilising the Aalen–Johansen plots to estimate the cumulative incidence of dementia and all-cause death in patients with SHS and those without. Daily cumulative incidence was determined by dividing the total number of new cases by the number of individuals at risk in each day of follow-up.

Sensitivity analyses were conducted to: (i) evaluate the 1-year risk of dementia in SHS patients without cardiovascular events prior the ischaemic stroke (e.g. AMI, HF, VFF and TTS); (ii) determine the 1-year risk for each type of dementia, prior to the ischaemic stroke; (iii) assess the risks of dementia and death at the second and third year after the ischaemic stroke; (iv) assess the 1-year risk of dementia associated with each SHS manifestation; (v) evaluate the 1-year dementia risk within relevant clinical subgroups (age <75 or ⩾75 years,^13^ males or females, those on oral anticoagulants (OAC), and those not on OAC); and (vi) account for the presence of a competing risks between dementia and all-cause death.

All analyses were executed within the TriNetX platform, which utilises both R and Python for data analysis. The R Survival library v3.2-3 was used for survival analyses, while propensity risk scores were estimated using logistic regression, implemented via the scikit-learn package in Python version 3.7. TriNetX does not impute or estimate clinical values to fill gaps in a patient’s record. All tests were two-tailed and statistical significance was defined as *p*-values < 0.05, indicating assuming a Type I error of less than 5% if the null hypothesis is true.

## Results

Overall, we included 907,203 patients with ischaemic stroke: 52,971 patients with SHS (mean age 66.6 ± 14.6 years, 42.2% females) and 854,232 patients without SHS (64.7 ± 15.4 years, 48.2% females).

Prior PSM, patients with SHS were slightly older, more likely to be males and had a higher cardiovascular burden compared to those patients without SHS (Table 1). Specifically, patients with SHS had a higher prevalence of cardiovascular risk factors, previous cardiovascular events and were more likely to receive cardiovascular treatments, including OAC and antiplatelets.

**Table 1.** Baseline characteristics comparison between patients with SHS and those without SHS, before and after propensity score matching.

Baseline characteristics	Before propensity score match			After propensity score match		
	Patients with SHS	Patients without SHS	ASD	Patients with SHS	Patients without SHS	ASD
	*n* = 52,971	*n* = 854,23		*n* = 52,970	*n* = 52,970	
Age, years (±SD)	66.6 ± 14.6	64.7 ± 15.4	0.128	66.6 ± 14.6	67.3 ± 14.3	0.052
Female, *n* (%)	22,358 (42.2)	411,811 (48.2)	0.121	22,358 (42.2)	21,690 (40.9)	0.026
White, *n* (%)	31,981 (60.4)	517,308 (60.6)	0.004	31,981 (60.4)	32,644 (61.6)	0.026
Black or African American, *n* (%)	9636 (18.2)	127,826 (15.0)	0.087	9635 (18.2)	9553 (18.0)	0.004
Asian, *n* (%)	1671 (3.2)	37,550 (4.4)	0.065	1671 (3.2)	1622 (3.1)	0.005
Arterial hypertension, *n* (%)	27,321 (51.6)	347,017 (40.6)	0.221	27,320 (51.6)	26,454 (49.9)	0.033
Atrial fibrillation, *n* (%)	10,013 (18.9)	78,203 (9.2)	0.283	10,012 (18.9)	9843 (18.6)	0.008
Diabetes mellitus, *n* (%)	15,296 (28.9)	167,270 (19.6)	0.218	15,295 (28.9)	14,975 (28.3)	0.013
Chronic kidney disease, *n* (%)	10,698 (20.2)	77,833 (9.1)	0.317	10,697 (20.2)	10,300 (19.4)	0.019
Obesity, *n* (%)	8052 (15.2)	84,027 (9.8)	0.163	8052 (15.2)	7593 (14.3)	0.024
Dyslipidaemia, *n* (%)	20,520 (39.0)	255,455 (29.9)	0.187	20,520 (38.7)	19,922 (37.6)	0.023
Chronic Ischaemic heart disease, *n* (%)	18,661 (35.2)	126,959 (14.9)	0.484	18,660 (35.2)	19,060 (36.0)	0.016
Chronic Heart failure, *n* (%)						
Systolic	4457 (8.4)	12,652 (1.5)	0.324	4456 (8.4)	4006 (7.6)	0.031
Diastolic	3137 (5.9)	13,649 (1.6)	0.229	3136 (5.9)	2934 (5.5)	0.016
Ischaemic stroke, *n* (%)	924 (1.7)	26,416 (3.1)	0.088	924 (1.7)	1078 (2.0)	0.021
Pulmonary embolism, *n* (%)	1514 (2.9)	13,421 (1.6)	0.088	1513 (2.9)	1442 (2.7)	0.008
Peripheral vascular disease, *n* (%)	5439 (10.3)	44,517 (5.2)	0.190	5439 (10.3)	5299 (10.0)	0.009
Sleep apnoea, *n* (%)	5473 (10.3)	56,145 (6.6)	0.135	5473 (10.3)	5217 (9.8)	0.024
Intracerebral haemorrhage, *n* (%)	763 (1.4)	17,746 (2.1)	0.048	763 (1.4)	660 (1.2)	0.017
Lipid-lowering drugs, *n* (%)	21,525 (40.6)	255,097 (29.9)	0.227	21,525 (40.6)	20,789 (39.2)	0.037
Beta-blockers, *n* (%)	23,221 (43.8)	234,115 (27.4)	0.348	23,220 (43.8)	22,823 (43.1)	0.015
Diuretics, *n* (%)	20,574 (38.8)	199,096 (23.3)	0.340	20,573 (38.8)	20,091 (37.9)	0.026
Antiarrhythmics, *n* (%)	17,084 (32.3)	180,696 (21.3)	0.242	17,083 (32.3)	16,254 (30.7)	0.034
Calcium channel blockers, *n* (%)	15,048 (28.4)	171,287 (20.1)	0.196	15,047 (28.4)	14,501 (27.4)	0.023
ACE inhibitors, *n* (%)	15,518 (29.3)	165,424 (19.4)	0.233	15,517 (29.3)	14,928 (28.2)	0.025
Angiotensin II inhibitors, *n* (%)	8189 (15.5)	92,904 (10.9)	0.136	8189 (15.5)	7718 (14.6)	0.025
Digoxin, *n* (%)	2872 (5.4)	14,925 (1.7)	0.199	2871 (5.4)	2611 (4.9)	0.022
Anticoagulant, *n* (%)	20,519 (38.7)	203,138 (23.8)	0.327	20,518 (38.7)	19,824 (37.4)	0.027
Antiplatelet, *n* (%)	21,427 (40.5)	239,242 (28.0)	0.265	21,426 (40.4)	20,726 (39.1)	0.027

ACE: Angiotensin-converting enzyme; ASD: Absolute Standardized mean Difference; SHS: Stroke-Heart Syndrome.

Following PSM, 52,970 patients were matched in each group, resulting in no significant differences between the two groups (Table 1). The median follow-up, after PSM, was 1013 days (IQR 946 days) in SHS patients and 1125 days (IQR 677 days) in patients without SHS.

The number of primary and secondary outcomes recorded during the 1-year follow-up is reported in Table 2. A total of 2027 (3.8%) new cases of dementia were recorded among patients with SHS compared to 1726 (3.3%) cases among those without SHS, HR 1.28, 95%CI 1.20–1.36. Additionally, the number of all-cause deaths recorded was 7636 (14.4%) in the SHS group and 3765 (7.1%) in the group without SHS, HR 2.22, 95%CI 2.14–2.31. Kaplan Meier curves for primary and secondary outcomes are reported in Supplemental Figures 2 and 3.

**Table 2.** Risk of primary and secondary outcomes in patients with SHS compared to those without SHS in different time windows after propensity score matching.

Time windows	Dementia				All-cause death			
	Patients with SHS (*N* = 52,970)	Patients without SHS (*N* = 52,970)	HR (95%CI)	χ^2^	Patients with SHS (*N* = 52,970)	Patients without SHS (*N* = 52,970)	HR (95%CI)	χ^2^
	*n* events (%)	*n* events (%)			*n* events (%)	*n* events (%)		
First year	2027 (3.8)	1726 (3.3)	1.28 (1.20–1.36)	17.080	7636 (14.4)	3765 (7.1)	2.22 (2.14–2.31)	51.326
First year*	250 (4.9) / 5126	157 (3.1) / 5126	1.73 (1.41–2.11)	11.450	735 (14.3) / 5126	287 (5.6) / 5126	2.77 (2.41–3.17)	29.854
First 31 days	489 (0.9)	332 (0.6)	1.51 (1.31–1.74)	0.121	2051 (3.9)	675 (1.3)	3.13 (2.87–3.41)	10.234
32 days – end of the first year	1742 (3.3)	1545 (2.9)	1.23 (1.15–1.32)	2.551	5628 (10.6)	3037 (5.7)	2.04 (1.96–2.14)	16.690
Second year	1366 (2.6)	1528 (2.9)	1.04 (0.97–1.12)	0.366	2810 (5.3)	2233 (4.2)	1.48 (1.40–1.56)	1.707
Third year	1156 (2.2)	1509 (2.8)	0.92 (0.85–0.99)	0.376	2194 (4.1)	1934 (3.7)	1.37 (1.29–1.46)	5.052

In each time window, propensity score matching was conducted de novo.

HR: Hazard Ratio; CI: Confidence Interval; SHS: Stroke-Heart Syndrome.

* Only in patients without previous cardiovascular events.

A high χ² suggests a greater deviation from the expected values, indicating a potential violation of the proportional hazard assumption. Conversely, a small χ² value indicates that the observed residuals closely match the expected values.

When analysing the proportional hazards assumption for the 1-year risk of primary and secondary outcomes in patients with SHS compared to those without SHS, we found that it was violated for both dementia (χ^2^ = 17.080, *p*-value for proportionality < 0.001) and all-cause death (χ^2^ = 51.326, *p*-value for proportionality < 0.001) (Table 2). When the follow-up was subdivided, we observed that the risk of dementia was significantly higher during the early phase in patients with SHS compared to those without SHS with no violation of the proportional hazards assumption (HR for early dementia: 1.51, 95%CI 1.31–1.74, χ² = 0.121, *p* for proportionality = 0.728). During the late phase, patients with SHS still showed a significantly increased risk of dementia compared to those without SHS, but the risk was of a lower magnitude than in the early phase. Again, no violation of the proportional hazards assumption was observed (HR for late dementia: 1.23, 95%CI 1.15–1.32, χ² = 2.551, *p* for proportionality = 0.110). Conversely, the risk of all-cause death exhibited a significant discrepancy with the expected HR in both the early (HR 3.13, 95%CI 2.87–3.41, χ² = 10.234, *p* for proportionality = 0.001) and late phases (HR 2.04, 95%CI 1.96–2.14, χ² = 16.690, *p* for proportionality < 0.001).

### Sensitivity analyses

The first sensitivity analysis confirmed the results of the main analysis, even when considering only patients without prior cardiovascular events. In patients with SHS, the 1-year risks of dementia and death were approximately 1.7–2.8 times the risk of those without SHS (Table 2). As for the main analysis, even in this case the proportional hazards assumption was not respected for both the primary and secondary outcomes (Table 2).

**Figure 1.**
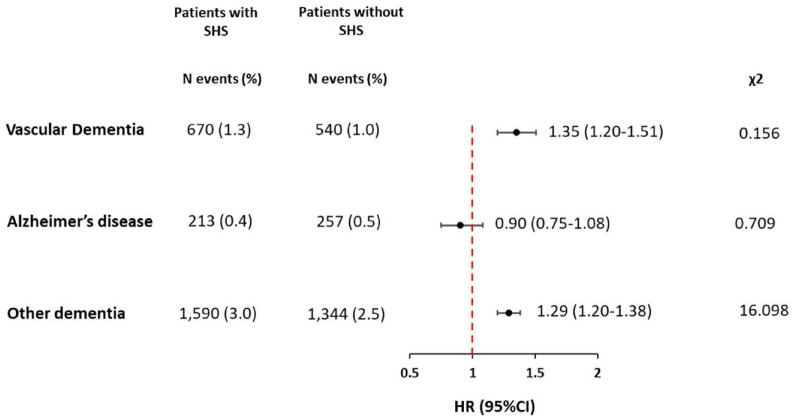
One-year risk of different types of dementia in patients with stroke-heart syndrome (*n* = 52,970) compared to those without stroke-heart syndrome (*n* = 52,970). CI: Confidence Interval; HR: Hazard Ratio; N: Number, SHS: Stroke-Heart Syndrome. Other dementia includes unspecified dementia and dementia in other diseases classified elsewhere. A high χ² suggests a greater deviation from the expected values, indicating a potential violation of the proportional hazard assumption. Conversely, a small χ² value indicates that the observed residuals closely match the expected values.

The second sensitivity demonstrated statistically significant differences in the 1-year risk for different types of dementia. In patients with SHS, the highest risk was for vascular and other types of dementia (Unspecified dementia and Dementia in other diseases classified elsewhere), whereas no significant association was found with Alzheimer’s disease (Figure 1). The assessment of the hazard proportionality assumptions showed that it was respected for vascular dementia and Alzheimer’s disease but was violated for other types of dementia (Figure 1).

The third sensitivity analysis showed that, with extended follow-up, the risk of dementia in patients with SHS decreased by approximately 10% during the second and third years, compared to patients without SHS, eventually becoming non-significant. However, there was no violation of the proportional hazards assumption in either the second or third year (Table 2).

Similarly, the risk of all-cause death decreased by approximately 20% annually, but it remained significantly higher in patients with SHS compared to those without SHS. Although the proportional hazards assumption was not violated during the second year, it became significant again in the third year of follow-up (Table 2).

The fourth sensitivity analysis, aimed at examining the risk of dementia for each specific manifestation of SHS, indicated that an increased risk of dementia was clear in cases involving AMI and HF, while TTS and VFF exhibited only a non-significant trend towards an increased dementia risk (Table 3). Conversely, the risk of all-cause mortality was significantly associated with all manifestations of SHS (Table 3). The proportional hazards assumption was respected for either dementia and all-cause death in patients with TTS or VFF, but it was violated in those with AMI or HF (Table 3).

**Table 3.** Risk of primary and secondary outcomes in patients with SHS compared to those without SHS, stratified by the type of cardiovascular events.

Type of post-stroke cardiovascular	Patients with SHS	Patients without SHS	HR (95%CI)	χ^2^
(*n* = number of patients for each group after PSM)	Number of events (%)	Number of events (%)		
AMI (*n* = 35,966)				
Dementia	1301 (3.6)	1093 (3.0)	1.28 (1.19–1.39)	5.928
All-cause death	4631 (12.9)	2969 (6.6)	2.12 (2.02–2.23)	46.101
HF (*n* = 21,621)				
Dementia	941 (4.4)	733 (3.4)	1.44 (1.31–1.59)	13.330
All-cause death	4004 (18.5)	1788 (8.3)	2.54 (2.40–2.69)	37.341
VFF (*n* = 1730)				
Dementia	45 (2.6)	3.8 (2.2)	1.35 (0.88–2.08)	1.590
All-cause death	338 (19.5)	105 (6.1)	3.67 (2.95–4.57)	0.182
TTS (*n* = 1312)				
Dementia	47 (3.6)	37 (2.8)	1.92 (0.88–2.08)	0.372
All-cause death	165 (12.6)	58 (4.4)	3.06 (2.27–4.13)	18.268

AMI: Acute Myocardial Infarction; CI: Confidence Interval; HF: Heart Failure; HR: Hazard Ratio; PSM: Propensity Score Matching; SHS: Stroke-Heart Syndrome; TTS: Takotsubo cardiomyopathy; VFF: Ventricular Flutter-Fibrillation.

A high χ² suggests a greater deviation from the expected values, indicating a potential violation of the proportional hazard assumption. Conversely, a small χ² value indicates that the observed residuals closely match the expected values.

**Figure 2.**
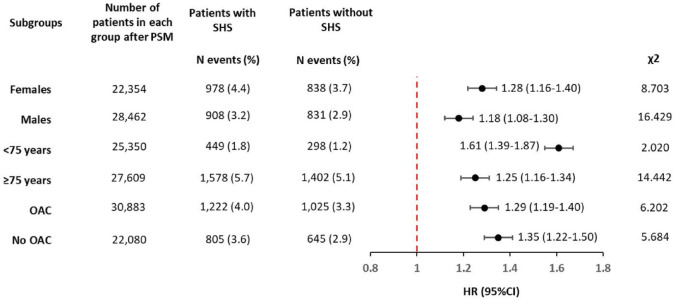
One-year risk of dementia in patients with stroke-heart syndrome compared to those without stroke-heart syndrome considering different clinically relevant subgroups. CI: Confidence Interval; HR: Hazard Ratio; PSM: Propensity Score Matching; N: Number; OAC: Oral Anticoagulants, SHS: Stroke-Heart Syndrome. A high χ² suggests a greater deviation from the expected values, indicating a potential violation of the proportional hazard assumption. Conversely, a small χ² value indicates that the observed residuals closely match the expected values.

The fifth sensitivity analysis demonstrated that the risk of dementia in patients with SHS compared to those without SHS was consistent across all subgroups analysed, irrespective of age (<75 or ⩾75 years), sex (male or female) and whether OAC were used (Figure 2). The proportional hazards assumption was not respected in most analyses, except for patients aged <75 years, where the risk of dementia was significantly higher compared to those aged ⩾75 years and the proportional hazards assumption was satisfied (Figure 2).

### Competitive risk analysis

**Figure 3.**
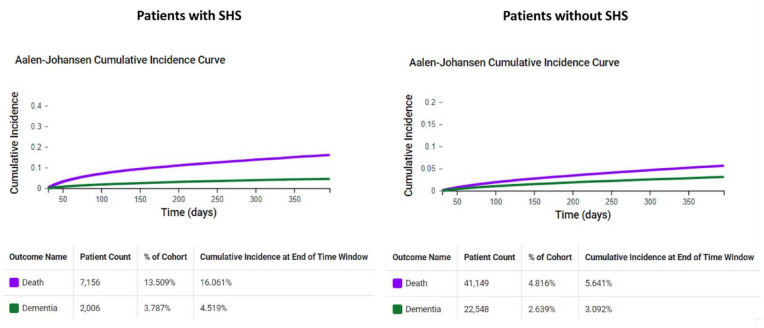
One-year Aalen-Johansen cumulative incidence curves for all-cause death and dementia. SHS: Stroke-Heart Syndrome.

In the analysis of daily cumulative risk for dementia and all-cause death among patients with and without SHS, we observed that the high daily cumulative incidence of all-cause death competes with the risk of developing dementia in both groups (Figure 3). This pattern was particularly pronounced in patients with SHS, who exhibited a daily cumulative incidence of all-cause death at 16.1%, nearly triple that of the 5.6% observed in patients without SHS. These findings suggest that the true risk of dementia in SHS survivors may be higher than the estimates presented in the main analysis.

## Discussion

In this retrospective, propensity score-matched analysis of a large cohort of patients with ischaemic stroke, we found that (i) patients with SHS had an increased risk of dementia and a higher risk of all-cause death at 1-year of follow-up compared to those without SHS; (ii) the increased risk of dementia was not constant over the time, with the highest risk during the first 31 days after the start of the follow-up; (iii) The increased risk of dementia in patients with SHS was consistent even when considering only patients without a history of cardiovascular events prior to the ischaemic stroke; (iv) The overall increased risk of dementia in patients with SHS was mainly due to vascular or other/mixed forms of dementia rather than Alzheimer’s disease; (v) Both the risk of dementia and all-cause death decreased over time, with the risk of dementia becoming non-significant during the second and third years of follow-up, while the risk of all-cause death remained statistically significant; (vi) All individual components of SHS were associated with a higher risk of both dementia and death, except for cases of TTS and VFF, which only demonstrated a non-significant increase in the risk of dementia. (vii) the increased 1-year risk of dementia observed was regardless of age, sex and OAC use. However, it was higher in those aged <75 years compared to those aged ⩾75 years.

Previous studies have demonstrated that both ischaemic stroke and cardiovascular disease are independently associated with an increased risk of dementia.^10,11,12,14–17^ In a meta-analysis of 1.9 million patients with prevalent stroke and 1.3 million patients with incident stroke, the authors found that the pooled HR for dementia was 1.69 (95%CI 1.49–1.92) for prevalent stroke and 2.18 (95%CI 1.90–2.50) for incident stroke.^15^ A prospective study on 23,572 patients from the US, followed for a median of 6.1 years, demonstrated that in those who experienced incident stroke (2.2%), global cognition declined faster compared to the pre-stroke period.^18^ Similarly, a large meta-analysis of 27 studies reported a pooled prevalence of post-stroke dementia of up to 18% at 1 year.^19^ Moreover, a community-based cohort of 1301 individuals aged ⩾75 years from Sweden, with a median follow-up of 9 years, showed that HF was associated with an increased risk of dementia (HR 1.84, 95%CI 1.35–2.51) and Alzheimer’s disease (HR 1.80, 95%CI 1.25–2.61).^10^ Similar results were reported in patients with AMI, where the risk of dementia was inversely related to the age of AMI onset^20^; in those with atrial fibrillation, where the risk was highest in individuals who developed this arrhythmia before the age of 65^21^; and in those who survived cardiac arrest.^22^

Ischaemic stroke may cause vascular cognitive impairment and dementia, through cerebral hypoperfusion that results from the acute vascular injury and can be heightened by the pre-existence of asymptomatic brain injuries due to cerebral small vessel disease.^23^ Thus, the cerebral hypoperfusion, which can result from both covert cerebrovascular disease and overt brain injury, is likely the primary mechanism leading to cognitive impairment in stroke patients.^24^ In this context, dysfunction in the brain-heart axis, associated with post-stroke AMI, HF, or arrhythmias, may impair the cardiac output and worsen cerebral hypoperfusion, contributing to cognitive impairment beyond the effects of brain infarcts.^25,26^ This hypothesis is supported by our main analysis, which found that the coexistence of both ischaemic stroke and early cardiovascular events was associated with an increased risk of dementia compared to ischaemic stroke alone. Our sensitivity analyses further revealed that this risk was the highest during the early follow-up phase and was primarily driven by vascular and mixed forms rather than Alzheimer’s disease. Additionally, the impact of SHS on dementia risk, was more pronounced in patients without previous cardiovascular events or in those under <75 years, where probably fewer pro-inflammatory confounders were present. We also observed a progressively reduced risk of dementia over the study period, which further support the pivotal role of the acute post-stroke neuronal injury in driving dementia risk, as the association become non-significant during the second and third year of follow-up. However, it should be noted that the declining risk of dementia over the study period may be partially attributable to the high risk of all-cause mortality in patients with SHS, which could have exacerbated the competing risk with dementia in later stages of follow-up and the progression of cardiovascular burden in patients without SHS – due to aging or the development of new cardiovascular risk factors or events – that may have increased the dementia risk over time in this group. Moreover, when hypothesising a direct effect of cerebral and cardiac hypoperfusion on the risk of dementia in patients with SHS, it should be considered that all these clinical conditions share common risk factors, including advanced age, smoking, obesity, hypertension, dyslipidaemia and diabetes.^27^ These risk factors have significant pro-atherosclerotic effects, which may contribute not only to ischaemic stroke and post-stroke cardiovascular complications but also to the risk of dementia itself.^28^ Myocardial injury in patients with acute ischaemic stroke and high atherosclerotic burden is associated with more extensive white matter lesions and greater global cognitive impairment.^29,30^ Additionally, in patients with ischaemic stroke and advanced generalised atherosclerosis, autonomic dysregulation may be facilitated.^31,32^ In this context, it is plausible that patients with SHS are more likely to develop vascular dementia due to the direct impact of vascular events on vulnerable brain tissue due to the preexisting atherosclerotic cerebral vasculopathy.

The high risk of adverse events in patients who develop dementia after ischaemic stroke or cardiovascular events highlights the need of methods to early identify patients at high risk of dementia. Early identification of SHS, through methods such as ECG or prolonged ECG monitoring or serial imaging with echocardiography or cardiac MRI, may help to identify patients at risk of vascular cognitive impairment and dementia. Additionally, dementia risk stratification in patients with SHS could be improved by incorporating brain MRI to detect those with white matter hyperintensities. Previous studies have shown that white matter hyperintensities are highly prevalent in patients with ischaemic stroke, atrial fibrillation, or HF and are associated with global cerebral hypoperfusion and poorer cognitive performance.^17,33,34^

Currently, no established disease modifying treatment exists for post-stroke dementia and treatments are focused on preventive therapies and risk factor modification.^35^ Some evidence suggests symptomatic benefits of acetylcholinesterase inhibitors, memantine, DL-3-n-butylphthalide and nootropics (e.g. cerebrolysin, actovegin and cortexin), which are available for use in various regions.^36–38^ However, the magnitude of these benefits and the quality of the available evidence are insufficient to support their recommendation for clinical use or to justify changes in practice guidelines at this stage.

Growing evidence has also shown that patients with ischaemic stroke treated with endovascular thrombectomy have better outcomes compared to those treated with thrombolysis or treated with standard medical management.^39^ Thus, more research is needed to investigate whether mechanical vascular destruction, by reestablishing cerebral blood flow, can be associated with a lower risk of dementia compared to other treatments. Moreover, no data are available on the potential use of pharmacological or mechanical treatments aimed at supporting cardiac function to improve cerebral perfusion during SHS. Regarding the optimisation of risk factors and comorbidities, this can be addressed through the ABCstroke pathway, an integrative approach to post-stroke management outlined in a position paper by the ESC (European Society of Cardiology) Council on Stroke.^40^ This approach is based on three key pillars: (i) avoiding stroke recurrence with optimal antithrombotic strategies; (ii) improving functional and psychological status through routine assessment of post-stroke cognitive and physical impairment, depression and anxiety and (iii) managing cardiovascular risk factors and comorbidities, along with promoting a healthy lifestyle.^41^ The benefits of this integrated approach were demonstrated in a prospective cohort of 2513 ischaemic stroke patients from the Athens Stroke Registry followed for a median of 30 months. In this study, full adherence to the ABCstroke pathway was associated with a reduced risk of stroke recurrence (HR: 0.61; 95%CI: 0.37–0.99), major adverse cardiovascular events (HR: 0.59; 95%CI: 0.39–0.88) and death (HR: 0.22; 95%CI: 0.12–0.41), making it a potentially beneficial tool in the context of SHS as well.^42^

### Strengths and limitations

To the best of our knowledge, this is the first study to investigate the association between the risk of incident dementia and SHS. The study is based on a large contemporary cohort of ischaemic stroke patients and the main results have been validated through several sensitivity analyses.

However, there are also several limitations. The retrospective and observational nature of the study makes it susceptible to selection bias and other unmeasured biases. As TriNetX network relies on administrative data, it may be prone to misclassification and could fail to capture outcomes occurring outside the network. In the PSM, we balanced the two populations based on the prevalence of cardiovascular disease, but not on its severity or specific type. This may have led to residual differences in baseline risk, which could have influenced the risk of incident dementia. Moreover, we focused solely on cardiovascular risk factors and medical treatments, potentially omitting other clinically important variables. Additionally, balancing for intrinsic characteristics of SHS, such as the high prevalence of cardiovascular diseases, may have biased the estimation of dementia risk, making it challenging to generalise the findings to the general population. Only a small subset of patients with ischaemic stroke had comprehensive data on stroke type and severity, limiting our ability to explore the relationship between these factors and the risk of incident dementia. As suggested by the competing risk analysis, the risk of incident dementia in both groups (patients with SHS and those without SHS) may be underestimated due to the high cumulative incidence of all-cause death. No data were available on compliance with medical treatments during the observation period, which prevented us from assessing the impact of vascular secondary prevention on the risk of dementia. The study is also limited by the inability to stratify the analysis according to the use of thrombolytics, or endovascular procedures. Lastly, we did not explore how social determinants of health or insurance-based healthcare systems affect access to healthcare and influence the risk of dementia.

## Conclusion

SHS is associated with an increased risk of dementia. Future studies are needed to develop innovative strategies for preventing complications associated with SHS and improving the long-term prognosis of these patients.

## Supplemental Material

sj-pdf-1-eso-10.1177_23969873241293573 – Supplemental material for Incident dementia in ischaemic stroke patients with early cardiac complications: A propensity-score matched cohort study Supplemental material, sj-pdf-1-eso-10.1177_23969873241293573 for Incident dementia in ischaemic stroke patients with early cardiac complications: A propensity-score matched cohort study by Tommaso Bucci, Sylvia E Choi, Christopher TW Tsang, Kai-Hang Yiu, Benjamin JR Buckley, Pasquale Pignatelli, Jan F Scheitz, Gregory YH Lip and Azmil H Abdul-Rahim in European Stroke Journal
