# Supplementary material for: Incident dementia in ischaemic stroke patients with early cardiac complications: A propensity-score matched cohort study
Source: Eur Stroke J. 2024 Nov 2;10(2):541–51. doi: 10.1177/23969873241293573 (PMC11558657; doi:10.1177/23969873241293573)
Supplement: sj-pdf-1-eso-10.1177_23969873241293573 – Supplemental material for Incident dementia in ischaemic stroke patients with early cardiac complications: A propensity-score matched cohort study [file sj-pdf-1-eso-10.1177_23969873241293573.pdf]

Incident dementia in ischaemic stroke patients with early cardiac complications: a propensity-score matched cohort study

Tommaso Bucci, Sylvia E. Choi, Christopher T.W. Tsang, Kai-Hang Yiu, Benjamin J.R Buckley, Pasquale Pignatelli, Jan F. Scheitz, Gregory Y. H. Lip, Azmil H. Abdul-Rahim

Supplementary materials (V2)

TriNetX Database

The TriNetX data are collected from member healthcare organizations (HCOs) and originate from their primary electronic health records (EHR) systems. Typically, an HCO is a large academic health centre with data from most of its affiliates. An HCO often includes multiple facilities, such as main and satellite hospitals. The data are stored on the TriNetX database via a physical server at the institution's data centre or a virtual hosted appliance. The TriNetX platform consists of a network of these appliances connected into a federated system, allowing queries to be broadcast to each appliance, with results subsequently collected and aggregated. Once the data are sent to the network, they are mapped to a standardised set of clinical terminologies and undergo a data quality assessment, including 'data cleaning' that rejects records that do not meeting TriNetX quality standards. The TriNetX database performs internal and extensive data quality assessments with every refresh based on conformance, completeness, and plausibility (http://doi.org/10.13063/2327-9214.1244). HIPAA (Health Insurance Portability and Accountability Act) compliance is achieved through deidentification of clinical patient data. Real-word data derived from TriNetX has been utilized for several scientific purposes, including clinical trial design and hypothesis generation studies (https://trinetx.com/real-world-resources/).

Available data types within the network include demographics, diagnoses (represented by ICD-10-CM codes), procedures (coded in ICD-10-PCS or CPT), and measurements (coded to LOINC). While the database provides extensive information about patients' diagnoses and procedures, other variables, such as socioeconomic and lifetime factors, are not comprehensively represented. The advantage of electronic health record data over insurance claim data is that both insured and uninsured patients are included. Moreover, electronic health record data better represent the diagnostic rates in the population presenting to healthcare facilities, providing an accurate account of the burden of specific diagnoses on healthcare systems.

One primary limitation of relying on diagnoses is that they do not account for undiagnosed patients who might have a condition but have not yet received medical support. Another general limitation of electronic health record data is that a patient may be seen at different HCOs for various components of their care. If one healthcare organization is not part of the federated network, part of their medical records may not be available. Using a network of healthcare organizations, rather than a single site, mitigates but does not fully eliminate this possibility.

Hazard Proportional assumption assessment.

To assess whether the proportional hazards assumption held in the Cox regression models, we applied a Chi-square (χ^2) test based on Schoenfeld residuals. These tests evaluate whether the relationship between the associated variables and the hazard function remains constant over time. The null hypothesis was that the effect of stroke-heart syndrome on the hazards of primary and secondary outcomes is constant over time. The χ^2 statistic quantifies the difference between the observed and expected Schoenfeld residuals. A larger χ^2 value suggests a greater deviation from the expected values, indicating a potential violation of the proportional hazard assumption. Conversely, a smaller χ^2 value implies that the observed residuals closely match the expected values, supporting the assumption. The p-value, derived from the χ^2 statistic, represents the probability of observing these deviations under the null hypothesis. A $p\text{-value} > 0.05$ indicates that the deviations are likely due to random variation, meaning the proportional hazards assumption holds. In contrast, a p-value < 0.05 suggests that the observed deviations are unlikely to be random, implying a violation of the proportional hazards assumption.

Figure 1. Flow chart of the study.

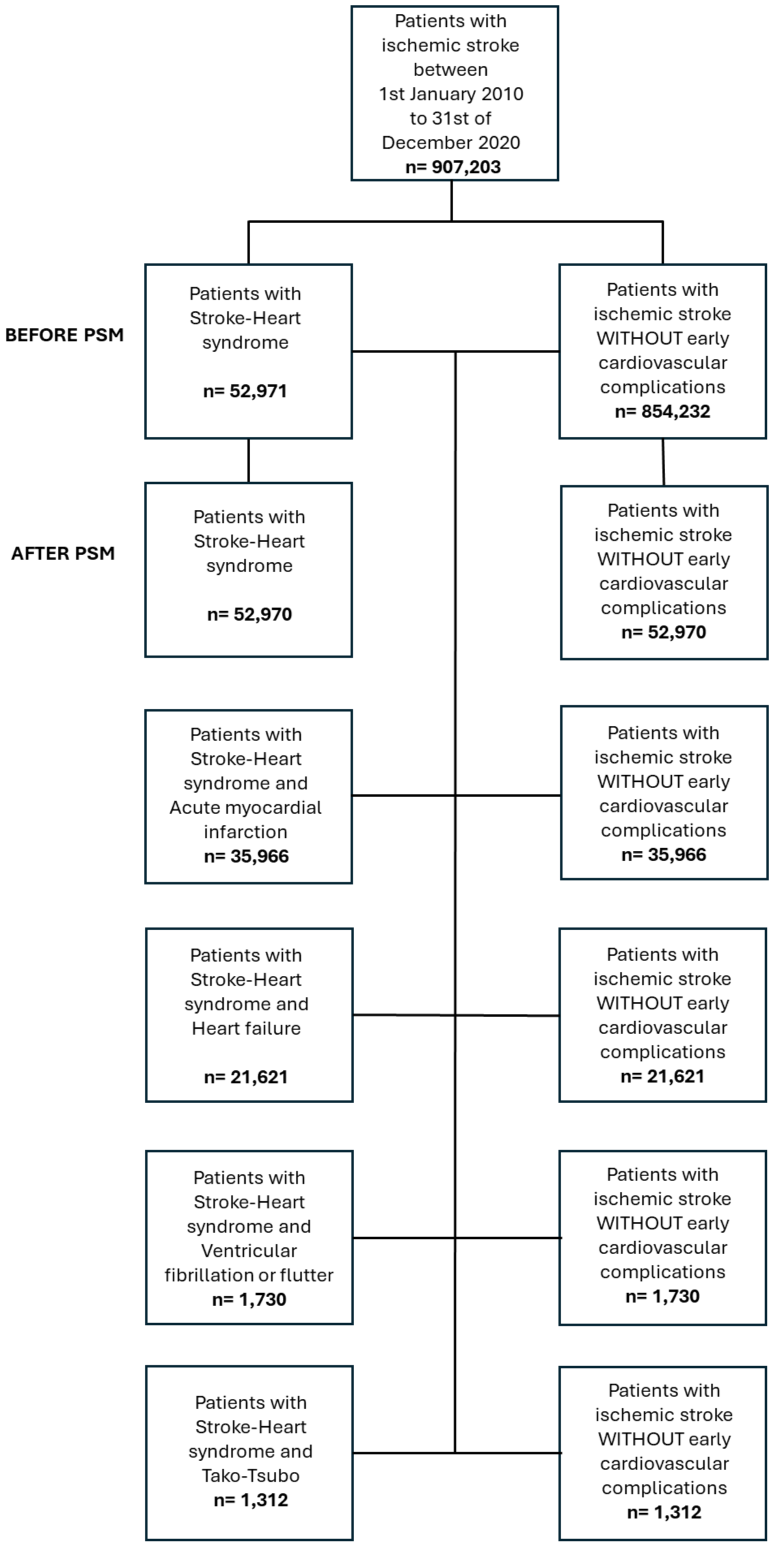

Supplementary Figure 2. Kaplan Meier curve for the risk of incident dementia in patients with Stroke Heart syndrome (purple line) and in those with ischemic stroke without early cardiovascular complications (green line).

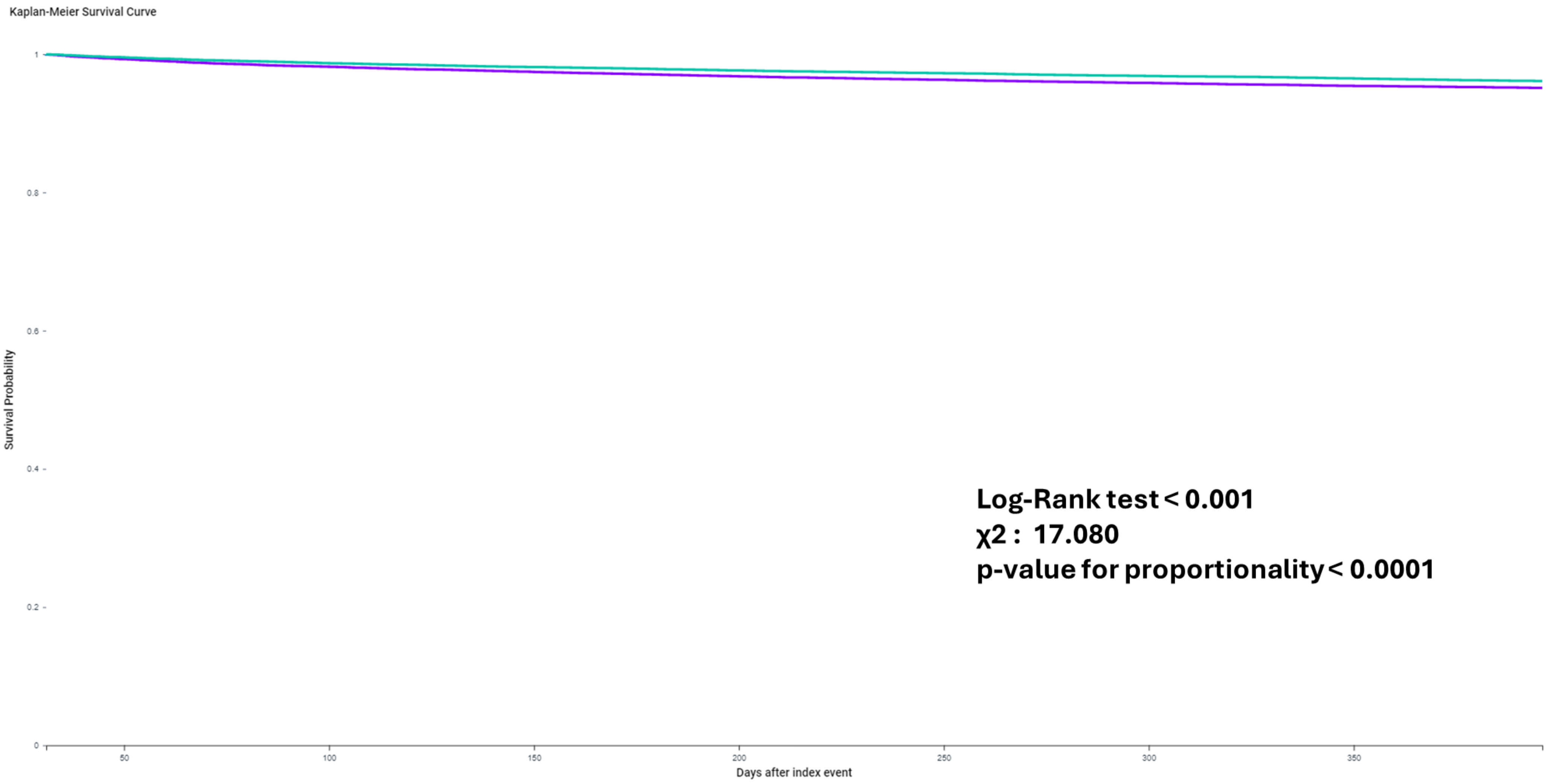

Supplementary Figure 3. Kaplan Meier curve for the risk of all-cause death in patients with Stroke Heart syndrome (purple line) and in those with ischemic stroke without early cardiovascular complications (green line).

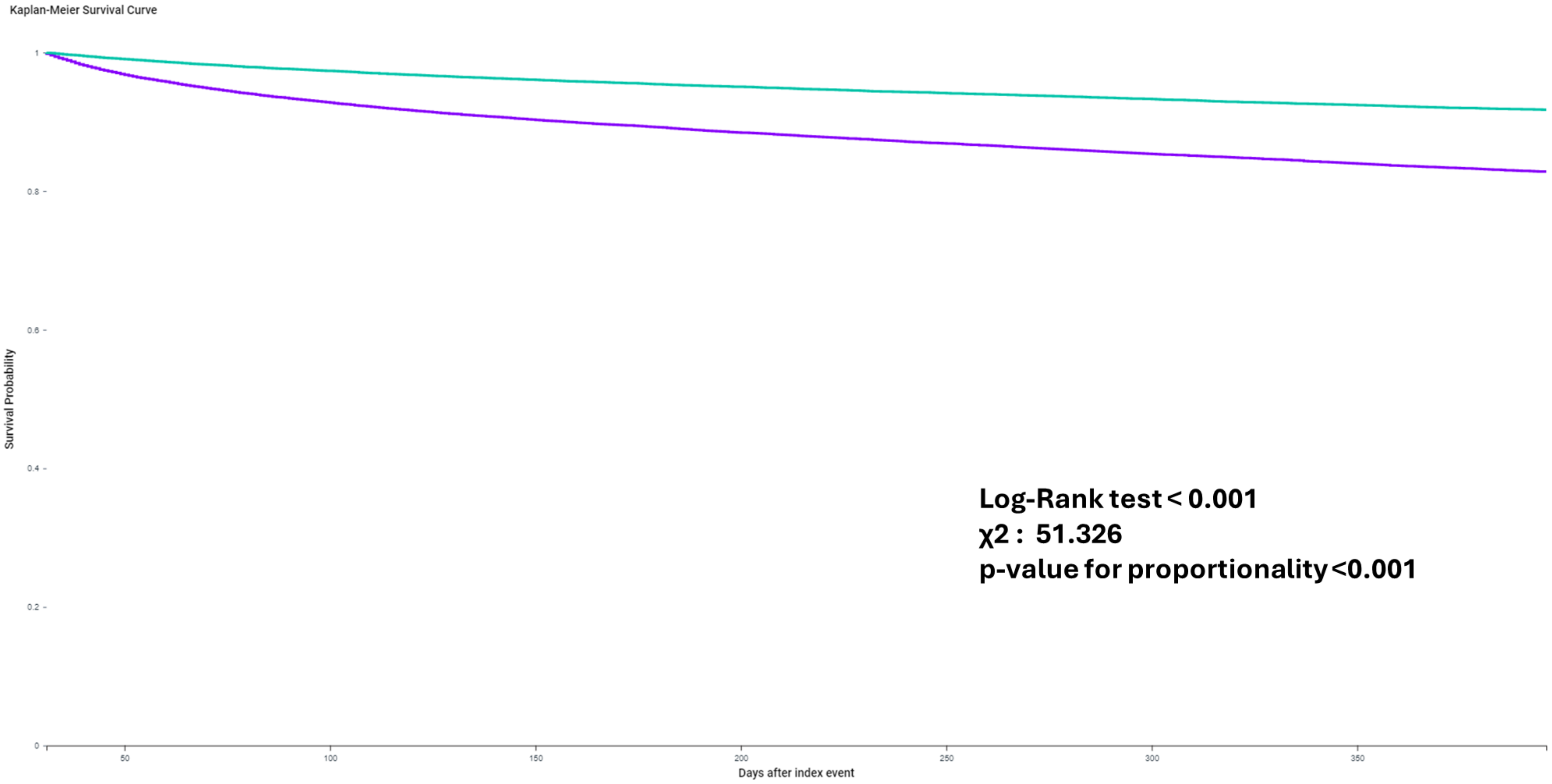

Supplementary Table 1. ICD-10-CM codes for inclusion and exclusion criteria in patients with Stroke-Heart syndrome and in those with stroke without early cardiovascular complications.

**Patients with Stroke-Heart syndrome**	
Inclusion criteria	1. First instance of ischemic stroke (ICD-10-CM I63) between 1st January 2010 to 31st of December 2020
AND one month after the first instance of stroke:	
Inclusion criteria	1) Takotsubo (ICD-10-CM I51.81)<br>2) Acute myocardial infarction (ICD-10-CM I21)<br>3) Acute heart failure (ICD-10-CM I50.21, 50.43, 50.23, 50.33, 50.31, 50.41)<br>4) Ventricular fibrillation and flutter (ICD-10-CM I49.0)
Exclusion criteria	1) Death
AND any time before the first instance of stroke:	
Exclusion criteria	1) Unspecified dementia (ICD-10-CM F03)<br>2) Alzheimer's disease (ICD-10-CM G30)<br>3) Vascular dementia (ICD-10-CM F01)<br>4) Dementia in other diseases classified elsewhere (ICD-10-CM F02)
**Patients with ischemic stroke without early cardiovascular complications**	
Inclusion criteria	1) First instance of ischemic stroke (ICD-10-CM I63) between 1st January 2010 to 31st of December 2020
AND one month after the first instance of stroke:	
Exclusion criteria	1) Takotsubo (ICD-10-CM I51.81)<br>2) Acute myocardial infarction (ICD-10-CM I21)

	3) Acute heart failure (ICD-10-CM I50.21, 50.43, 50.23, 50.33, 50.31, 50.41)<br>4) Ventricular fibrillation and flutter (ICD-10-CM I49.0)<br>5) Death
AND any time before the first instance of stroke:	
Exclusion criteria	1) Unspecified dementia (ICD-10-CM F03)<br>2) Alzheimer's disease (ICD-10-CM G30)<br>3) Vascular dementia (ICD-10-CM F01)<br>4) Dementia in other diseases classified elsewhere (ICD-10-CM F02)

Supplementary Table 2. ICD-10-CM codes for the one-year risk of all-cause death and dementia.

All-cause death	• Deceased (TriNetX variable)
Dementia	• Unspecified dementia (ICD-10-CM F03)<br>• Alzheimer's disease (ICD-10-CM G30)<br>• Vascular dementia (ICD-10-CM F01)<br>• Dementia in other diseases classified elsewhere (ICD-10-CM F02)
